# Supplementary material for: Reconciling Mining with the Conservation of Cave Biodiversity: A Quantitative Baseline to Help Establish Conservation Priorities
Source: PLoS One. 2016 Dec 20;11(12):e0168348. doi: 10.1371/journal.pone.0168348 (PMC5173368; doi:10.1371/journal.pone.0168348)
Supplement: S1 Dataset — (ZIP) [file pone.0168348.s002.zip › Taxa/Serra Norte/SN_2013/N4WS/Lista N4WS-29.pdf]

CAVIDADE N4WS-0029

Classe	Ordem	Fam/Outros	Gên/Outros	Espécie	S-U	U-S	Zon.
Arachnida	Acari	(Parasitengonina)		sp.2		X	E
Arachnida	Amblypygi	Charinidae	*Charinus*	jovem		1	E
Arachnida	Araneae	Filistatidae		sp.1		X	E
Arachnida	Araneae	Ochyroceratidae		jovem		X	E
Arachnida	Araneae	Pholcidae		jovem	X	X	E
Arachnida	Araneae	Theridiidae		jovem		X	E
Arachnida	Araneae	Theridiosomatidae	*Plato*	sp.1		X	E
Arachnida	Opiliones	Escadabiidae		sp.1	X		E
Arachnida	Pseudoscorpiones	Chthoniidae	*Pseudochthonius*	sp.1	X		E
Arachnida	Pseudoscorpiones	Olpiidae		sp.1		X	E
Entognatha	Collembola	Isotomidae		sp.1		X	E
Entognatha	Collembola	Paronellidae		sp.1	X	X	E
Insecta	Blattodea			jovem	1		E
Insecta	Diptera	Sciaridae	*Bradysia*	sp.		X	E
Insecta	Heteroptera	Lygaeidae	Rhyparochrominae	sp.1		X	E
Insecta	Heteroptera	Reduviidae	Emesinae	sp.1		1	E
Insecta	Homoptera	Cixiidae		sp.2		X	E
Insecta	Hymenoptera	Chalcidoidea		sp.1		X	E
Insecta	Isoptera			sp.		X	E
Insecta	Lepidoptera	Noctuoidea		sp.4		X	E
Insecta	Neuroptera	Myrmeleontidae		jovem		4	E
Insecta	Orthoptera	Phalangopsidae	*Phalangopsis*	sp.		1	E
Insecta	Psocoptera	Psocomorpha		jovem	X		E
Insecta	Psocoptera	Psyllipsocidae		jovem		X	E
Insecta	Thysanoptera	Thripidae		sp.1		X	E
Turbellaria	Tricladida			sp.1		X	E
Amphibia	Anura	Strabomantidae	*Pristimantis*	cf. *fenestratus*	1	1	E
